# Multimodal imaging reveals a role for Akt1 in fetal cardiac development

**DOI:** 10.1002/phy2.143

**Published:** 2013-11-07

**Authors:** Katrien Vandoorne, Moriel H Vandsburger, Karen Weisinger, Vlad Brumfeld, Brian A Hemmings, Alon Harmelin, Michal Neeman

**Affiliations:** 1 Biological Regulation, Weizmann Institute of ScienceRehovot, Israel; 2 Biomedical engineering, Eindhoven University of TechnologyEindhoven, The Netherlands; 3 Chemical Research Support, Weizmann Institute of ScienceRehovot, Israel; 4 Friedrich Miescher Institute for Biomedical ResearchBasel, Switzerland; 5 Veterinary Resources, Weizmann Institute of ScienceRehovot, Israel

**Keywords:** Akt1, echocardiography, fetus, heart, mice

## Abstract

Even though congenital heart disease is the most prevalent malformation, little is known about how mutations affect cardiovascular function during development. Akt1 is a crucial intracellular signaling molecule, affecting cell survival, proliferation, and metabolism. The aim of this study was to determine the role of Akt1 on prenatal cardiac development. In utero echocardiography was performed in fetal wild-type, heterozygous, and Akt1-deficient mice. The same fetal hearts were imaged using ex vivo micro-computed tomography (*μ*CT) and histology. Neonatal hearts were imaged by in vivo magnetic resonance imaging. Additional ex vivo neonatal hearts were analyzed using histology and real-time PCR of all three groups. In utero echocardiography revealed abnormal blood flow patterns at the mitral valve and reduced contractile function of Akt1 null fetuses, while ex vivo *μ*CT and histology unraveled structural alterations such as dilated cardiomyopathy and ventricular septum defects in these fetuses. Further histological analysis showed reduced myocardial capillaries and coronary vessels in Akt1 null fetuses. At neonatal age, Akt1-deficient mice exhibited reduced survival with reduced endothelial cell density in the myocardium and attenuated cardiac expression of vascular endothelial growth factor A and collagen I*α*1. To conclude, this study revealed a central role of Akt1 in fetal cardiac function and myocardial angiogenesis inducing fetal cardiomyopathy and reduced neonatal survival. This study links a specific physiological phenotype with a defined genotype, namely Akt1 deficiency, in an attempt to pinpoint intrinsic causes of fetal cardiomyopathies.

## Introduction

Congenital heart disease affects almost 1% of human births and is the most prevalent malformation in the fetal and neonatal period (Hoffman et al. 2004). Significant progress has been made over the past decade in elucidating genetic and environmental factors causing these malformations in animal models and clinical studies (Andelfinger 2008). Functional analysis of fetal hearts in utero would provide a powerful noninvasive tool to associate a given genotype not only with morphological changes, but also with functional alterations. Characterizing fetal circulatory physiology using ultrasound biomicroscopy has been initiated in the past decade (Aristizabal et al. 1998; Pedra et al. 2002; Phoon and Turnbull 2003), allowing mechanistic understanding of cardiac malformations. The use of magnetic resonance imaging (MRI) for in utero cardiac imaging has been demonstrated (Nieman et al. 2009), yet reconstruction of the MR images remains challenging due to embryonic motion.

Akt, a protein kinase, plays a crucial role in processes such as cell survival, growth and proliferation, metabolism, and organogenesis (Yang et al. 2003; Plaks et al. 2010; Vandoorne et al. 2010). Of the three Akt isoforms, Akt1 plays an essential role in the induction of angiogenesis as a downstream effector of vascular endothelial growth factor (VEGF-A) through the phosphoinositide 3-kinase -Akt signaling pathway (Chen et al. 2005). Previous studies including those from our lab have described reduced angiogenesis in Akt1 deficient placenta and bone (Yang et al. 2003; Plaks et al. 2010; Vandoorne et al. 2010). Expression and phosphorylation levels of Akt isoforms in the heart of lacking the Akt1 gene (*Akt1*^*−/−*^) showed that Akt1 is the main isoform in the heart (Chang et al. 2010). Furthermore, *Akt1*^*−/−*^ fetuses exhibit ventricular dilatation with ventricular septum defects (VSDs) on histological sections, yet apoptosis in Akt1^*−*/*−*^ fetuses has not been found different than wild-type embryos (Chang et al. 2010). These defects in *Akt1*^*−/−*^ fetuses presumably lead to impaired neonatal cardiac function as detected by echocardiographic examination due to enhanced p38MAPK activation (Chang et al. 2010). Consequently, it has been assumed that *Akt1*^*−/−*^ mice exhibit enhanced postnatal mortality (Cho et al. 2001; Yang et al. 2003; Plaks et al. 2010) due to progressive contractile failure of neonatal *Akt1*^*−/−*^ hearts (Chang et al. 2010).

We hypothesized that mouse fetuses systemically lacking Akt1 might display early cardiac failure, not detected on postmortem histology. We sought to characterize blood flow velocities, to achieve a better mechanistic appreciation of fetal cardiac failure, by applying multi-parameter analyses of in utero fetuses with echocardiography. The complex interaction between VEGF-A and Akt (Chen et al. 2005), led us to also assess myocardial angiogenesis in *Akt1*^*−/−*^ mice during development.

## Material and Methods

### Animals

Animal experiments were approved by the Weizmann Institutional Animal Care and Use Committee following US National Institutes of Health, European Commission and Israeli guidelines. For in utero echocardiography, 14 heterozygous (*Akt1*^*+/−*^) females were mated with 3 *Akt1*^*+/−*^ males, yielding in total 24 wild-type (*Akt1*^*+/+*^), 31 *Akt1*^*+/−*^, 18 *Akt1*^*−/−*^ mice on a C57bl/6 background (Yang et al. 2003) at two fetal ages, namely embryonic day (E) 16.5 and E18.5. After echocardiography imaging sessions, dams and fetuses were sacrificed using an overdose of intraperitoneal (IP) pentobarbital, a sample was taken from the tail of each fetus for genotyping, and the fetal hearts were prepared for either ex vivo *μ*CT imaging or histological analysis. Neonatal *Akt1*^*+/+*^, *Akt1*^*+/−*^ and *Akt1*^*−/−*^ mice were generated through heterozygote breeding pairs and age-matched littermates were used. Furthermore, 9 *Akt1*^*+/+*^, 8 *Akt1*^*+/−*^, 7 *Akt1*^*−/−*^ neonates, of which 2–3 per genotype were in vivo imaged with cardiac MRI, were sacrificed at postnatal day 3 (P3) for histology, or for quantitative real-time polymerase chain reaction (qRT-PCR). Mice were housed at 21 ± 1°C, 40–50% humidity, on a 12 h light–dark cycle, with ad libitum access to water and standard rodent food.

### Genotyping

Tail samples were lysed overnight at 55^°^C in the presence of proteinase-K and DNA lysis buffer (Sigma-Aldrich, Saint-Louis, MO), and then denatured by heating at 85°C for 45 min. The resultant supernatant was used in a PCR reaction to identify the genotype of the mice. The following primers were used in the PCR reaction: (1) *Akt1* forward- 5′-TTGTCTCACGTGCTTTCTCG-3′, (2) *Akt1* reverse – 5′-5′-CCTGCTGGGTCAGTAAAGA-3′, (3) LacZ-cassette – 5′-GCGGATTGACCGTAATGG-3′; The amplified PCR product was subjected to agarose-gel electrophoresis and visualized using an infrared detection system.

### In utero echocardiography

For in utero echocardiography 14 Akt1^+/*−*^ females were mated with Akt1^+/*−*^ males. Pregnant Akt1^+/*−*^ dams were imaged at embryonic days E16.5 (*n* = 8) and E18.5 (*n* = 6), yielding, respectively, 17 and 8 Akt1^*−*/*−*^, 15 and 16 Akt1^+/*−*^, and 9 and 9 Akt1^+/+^ fetuses measured for, respectively, E16.5 and E18.5. These ages were chosen to avoid nucleated red blood cells in the blood of younger fetuses which contain hyperechogenic blood that hinders delineation of myocardial borders at echocardiography. In utero fetal echocardiography was performed using a Vevo 770 high-resolution ultrasound system with a RMV770B scanhead (Visualsonics, Toronto, Canada).

### Animal handling

Dams were anesthetized (isoflurane; induction 3.5%, maintenance 1.25–2%, in oxygen). Fur of the mother's abdomen was removed and prewarmed ultrasound transmission gel was applied over the entire abdomen. Body temperature was kept at 36–37°C using a preheated platform, and heart rate (HR) at 400–550 beats per minute (bpm) measured with ECG leads, by adjusting isoflurane level. Following the echocardiography imaging session, dams and fetuses were sacrificed using an overdose of IP pentobarbital, a sample was taken from the tail of each fetus for genotyping, and the fetal hearts were prepared for ex vivo *μ*CT imaging or histological analysis. Care was taken during imaging and sacrifice to accurately record the position of each fetus such that the genetic, echocardiographic and histological data would be paired correctly.

### Imaging protocol

In order to image fetal cardiac structure, B-mode (lateral resolution: 115 *μ*m) was performed through the mother's abdominal wall. For each measurement, at least three consecutive cardiac cycles were measured and averaged. To decrease experimental bias, all of the echocardiographic measurements were performed without knowing the genotype. Echocardiographic measurements are sensitive to the imaging planes used for ultrasound data acquisition. As echocardiographic measurements depend on fetal position, not all echocardiographic parameters could be acquired for each fetus. The left side of the fetuses was determined using B-mode imaging to determine the left ventricle (LV). Measurements were done using M-mode and Pulsed-wave (PW) Doppler mode. Inflow and outflow blood velocities at the mitral valve were registered using the PW Doppler mode. Echocardiography was performed as described before in C57bl/6 fetal hearts (Yu et al. 2008).

### Image analysis

From inflow waveforms the E peak (early) and A peak (active) were measured to calculate the E/A ratio. For outflow of the LV, isovolumetric relaxation time (IRT), isovolumetric contraction time (ICT), and time (ET) were measured. The myocardial performance index (MPI), clinically referred to as Tei index, was calculated as:


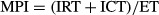


Fractional shortening (FS) was calculated from M-mode images (axial resolution = 55 *μ*m) by measuring left ventricular internal diameter (LVID) during systole (LVIDs) and diastole (LVIDd) as:


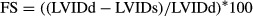


As these ultrasound measurements were two-dimensional, we did not derive three-dimensional (3D) parameters.

### Ex vivo *μ*CT of fetal hearts

Ex vivo *μ*CT was performed at E16.5 and E18.5 hearts (*n* = 4 for each genotype, each age). Samples were fixed, treated with Lugol's solution (Degenhardt et al. 2010), and examined using a Micro-XCT 400 system (Xradia, Pleasanton, CA). A total of 300 projection images were taken with magnification 4×, exposure time of 5 sec and voxel size 4.6 *μ*m. Image analyses, including 3D segmentation, 3D volume rendering, and volume measurements, were performed using Avizo software (VSG International, Burlington, MA).

### Neonate cardiac magnetic resonance imaging

Neonatal mice at postnatal day 3 (P3) (*Akt1*^*+/+*^: n=2; *Akt1*^*+/-*^: n=3; *Akt1*^*-/-*^: n=1) were imaged in vivo before euthanasia for ex vivo histology. Cardiac MRI was performed on a 9.4T using a linear resonator for excitation and an actively decoupled 2-cm surface coil for detection (Bruker, Rheinstetten, Germany). Mice were anesthetized (isoflurane; induction 3.5% in oxygen in a box, maintenance 1.25% in oxygen inhaled through a nose cone. Body temperature of the animals was kept between 36 and 37°C using a warming water blanket (Bruker, Germany) and HR was monitored using a fiber-optic system (Small animal Inc., Stony Brook, NY), and kept at 300–400 bpm. Gradient echo FLASH sequence was used with retrospective gating, acquiring 10 frames per cardiac cycle for both short-axis and (two-chamber and four-chamber) long-axis images (Intragate, Bruker, Germany). Imaging parameters for short axis: four to six slices from base to apex; Slice thickness: 0.8; Interslice distance: 1 mm; flip angle 10°; TR/TE 6.2716/2.9106 msec; number of repetitions 80; matrix 256 × 256; field of view (FOV) 20 × 20 mm; acquisition time 7 min 8.1 sec. Imaging parameters for long axis: Slice thickness: 1 mm; flip angle 10°; TR/TE 5.97/2.878 msec; number of repetitions 300; matrix 256 × 256; FOV 20 × 20 mm; acquisition time 4 min 2.829 sec.

### Histology and Fluorescence Microscopy

Hearts were fixed, embedded in paraffin, and sectioned serially at 4 *μ*m thickness (E16.5 hearts [*n* = 4 for each group] and P3 hearts [*n* = 3–4 for each group]). Tissue sections were stained with hematoxylin and eosin (H&E). Light microscopic images were taken with a Nikon E800 camera (Nikon, Tokyo, Japan) and quantitative histological analysis was performed using ImageJ. Coronal H&E cardiac sections (three sections/animal) were prepared from the middle of the heart. For coronary blood vessels, the number of vessels larger than 90 μm was calculated in the fetal heart close to the epicardium. For myocardial capillaries, the number of vessels within one high-power field (HPF) on myocardial sections was calculated. Endothelial cells in the myocardium at P3 were visualized by using *Bandeiraea simplicifolia* isolectin B4 (lectin-fluorescein isothiocyanate [FITC]) staining (1:100; Sigma*-*Aldrich L2895) (May et al. 2008). Fluorescent images were acquired on a Zeiss Axio observer microscope (Zeiss, Jena, Germany) equipped with a fluorescence illuminator and an Olympus DP72 camera using the Cell∧A camera-controlling software (Olympus, Center Valley, PA). The percent area green (lectin-FITC) fluorescent staining in each field of view was calculated using ImageJ software; the same threshold was applied for all samples (three to five sections/animal).

### RNA extraction and QRT-PCR

Quantitative real-time polymerase chain reaction was performed on hearts excised from P3 mice. After euthanasia (as previously described), hearts were rapidly excised, frozen, and RNA was isolated using the NucleoSpin RNA II kit (Macherey-Nagel, Duren, Germany). Total RNA (200 ng) was used for reverse transcription using SuperScript II RNase H-reverse (Invitrogen, Carlsbad, CA). QRT-PCR was performed using the StepOnePlus QRT-PCR system (Applied Biosystems, Carlsbad, CA) with the following primers: mouse VEGF-A (Forward primer: 5′-CATTCCTGGCCCTGAGTCAA and Reverse primer: 5′-GGTTGGAACCGGCATCTTTA); mouse collagen I*α*1 (COL1A1; Forward primer: 5′-GAAACCCGAGGTATGCTTGA and Reverse primer: 5′- GACCAGGAGGACCAGGAAGT). The transcription level was normalized to the expression of Hypoxanthine Phosphoribosyltransferase (HPRT, Forward primer: 5′-GGTCCTTTTCACCAGCAA and Reverse primer: 5′-GCAGTACAGCCCCAAAATGG). Each sample ran at least in duplicates.

### Statistical analysis

All data are presented as mean ± SEM. Statistical analysis was performed with JMP (JMP 6.0.3, SAS Institute, Cary, NC). All data were analyzed using one-way analyses of variance (ANOVA) followed by Tukey HSD All-Pairwise Comparisons test. Differences were considered significant at *P* < 0.05.

## Results

### Abnormal blood flow pattern at the mitral valve and reduced contractile function of Akt1-deficient fetuses

**Figure 1:**
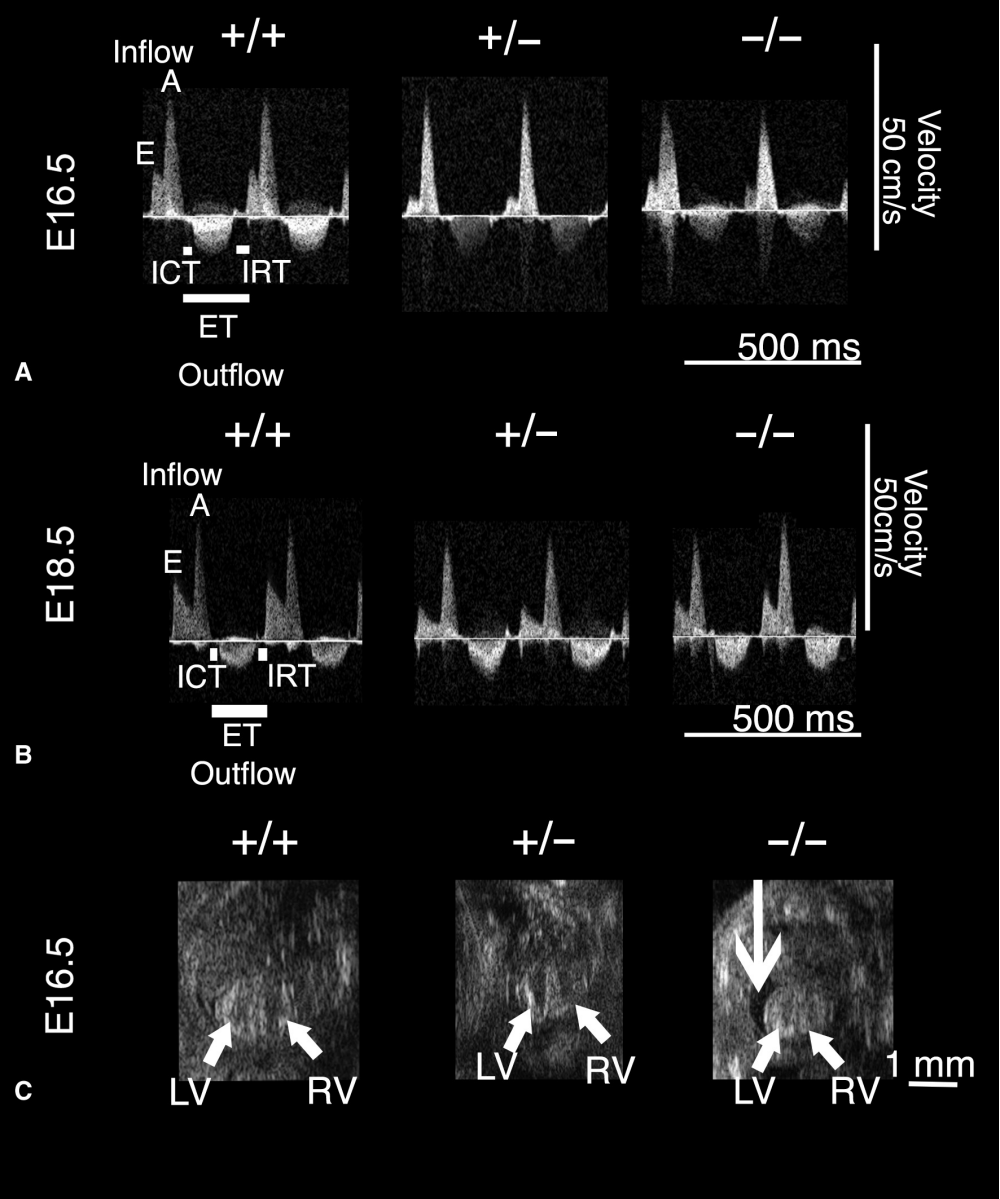
Akt1 disruption affected fetal cardiac function at embryonic day (E) 16.5 and E18.5. (A and B) In utero PW Doppler-positive blood velocities represent the inflow with early filling resulting in the E wave, and active filling (A wave), whereas negative peaks stand for outflow velocities from the LV encompassing ICT, IRT and ET. Representative E16.5 (A) and E18.5 (B) Doppler waveforms of *Akt1*^*+/−*^ and *Akt1*^*−/−*^ fetuses showed decreased E peak, resulting in a reduced E/A ratio compared to *Akt1*^*+/+*^. (C) B-mode ultrasound biomicroscopy (UBM) images depicted the fetal left ventricle (LV) and right ventricle (RV). Mild pericardial effusion (arrow), described for fetal heart failure, was found in some *Akt1*^*−/−*^ fetuses. +/+, *Akt1*^*+/+*^; +/*−*, *Akt1*^*+/−*^; *−*/*−*, *Akt1*^*−/−*^.

In utero fetal ultrasound detected similar fetal HRs for all genotypes. Blood PW waveforms at the mitral valve for fetal E16.5 and E18.5 hearts, revealed a reduced early (E) ventricular filling velocity in *Akt1*^*+/−*^ and *Akt1*^*−/−*^ fetuses compared to *Akt1*^*+/+*^ fetuses (Fig. 1A and B). Consequently, the E/A ratio, which assesses compliancy of the heart as well as diastolic function, was reduced in *Akt1*^*+/−*^ fetuses at E16.5 and in *Akt1*^*−/−*^ fetuses at E16.5 and E18.5 compared to *Akt1*^*+/+*^ fetuses. This is consistent with a restrictive filling pattern in *Akt1*^*−/−*^ fetuses (Table 1). E/A ratio increased from E16.5 to E18.5 in all genotypes, as described before (Yu et al.^2008^). MPI values, combining both systolic and diastolic cardiac performance, were significantly increased in *Akt1*^*+/−*^ and *Akt1*^*−/−*^ at E16.5 and E18.5 compared to *Akt1*^*+/+*^, suggesting reduced cardiac function. FS decreased significantly in *Akt1*^*−*/*−*^ fetuses at E16.5 and E18.5 and in *Akt1*^+/*−*^ fetuses at E16.5 (Table 1). Moderate pericardial effusion, previously described for fetal heart failure (Ranger et al. 1998), was seen in 3 of 17 E16.5 *Akt1*^*−/−*^ fetuses (Fig. 1C) and 1 of 8 E18.5 *Akt1*^*−/−*^ fetuses. We could not observe any dead fetuses. Thus, *Akt1*^*−/−*^ fetuses exhibited abnormal blood flow velocities and reduced cardiac function during systole and diastole.

**Table 1:** Echocardiographic measurements

Fetal age	E16.5			E18.5		
		
Genotype Akt1	+/+	+/*−*	*−*/*−*	+/+	+/*−*	*−*/*−*
n fetuses PW doppler	17	15	9	8	16	9
Heart rate (bpm)	246 ± 8	217 ± 8	235 ± 18	231 ± 19	252 ± 11	224 ± 19
E/A ratio	0.39 ± 0.01	0.34 ± 0.01*	0.29 ± 0.02*	0.45 ± 0.03	0.39 ± 0.02	0.36 ± 0.02*
Isovolumetric contraction time (msec)	18.7 ± 0.8	27.9 ± 1.4*	25.2 ± 2.5*	16.2 ± 1.2	21.5 ± 1.3	24.7 ± 2.9*
Isovolumetric relaxation time (msec)	29.0 ± 1.4	32.5 ± 1.0	35.4 ± 1.3*	28.0 ± 1.4	28.0 ± 1.2	30.0 ± 1.8
Ejection time (msec)	103.5 ± 4.0	112 ± 2.6	106.4 ± 6.7	115.8 ± 7.7	99 ± 3.2	102.6 ± 8.9
Myocardial performance index	0.46 ± 0.01	0.54 ± 0.02**	0.52 ± 0.03**	0.40 ± 0.02	0.50 ± 0.02**	0.53 ± 0.02**
n fetuses M-mode	12	15	7	8	13	6
Fractional Shortening (%)	42.7 ± 2.7	33.6 ± 2.5*	29.8 ± 3.7*	46.1 ± 2.8	43.0 ± 1.9	35.7 ± 2.4*

A +/+, *Akt1^+/+^*; +/*−*, *Akt1^+/−^*; *−*/*−*, *Akt1^−/−^*.

* *P* < 0.05;

** *P* < 0.01 vs. *Akt1^+/+^*.

### Left ventricular volume and fetal weight are reduced in Akt1-deficient fetuses

**Figure 2:**
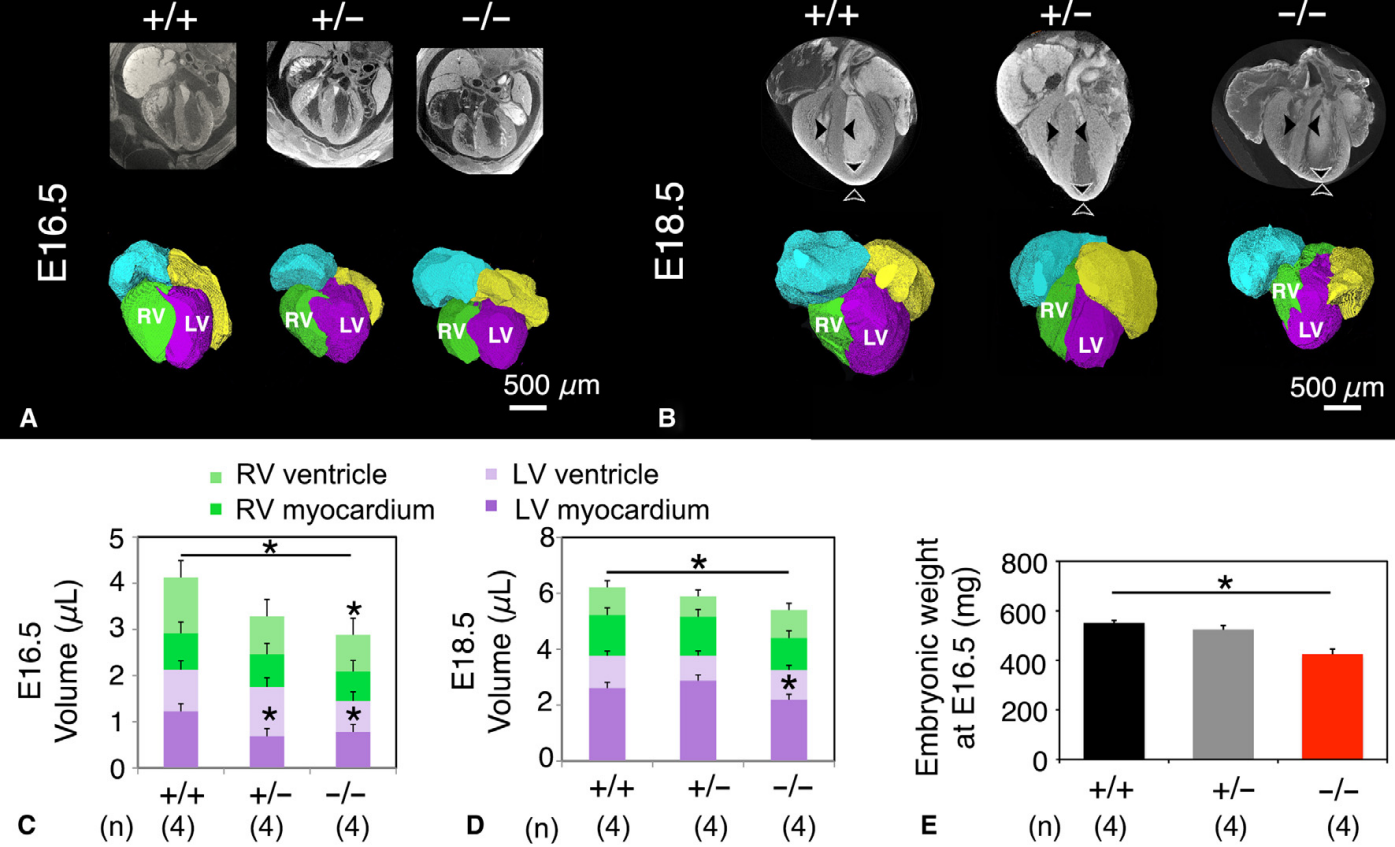
*Akt1*^*−/−*^ fetuses displayed smaller hearts and reduced fetal weight. (A and B) Ex vivo *μ*CT images and volume-rendered hearts revealing reduced size with a thinner myocardium and ventricular septum with a dilated morphology in *Akt1*^*−/−*^ mice (B, arrowheads). (C and D) Quantification of volumes of fetal heart at E16.5 (C) and E18.5 (D), illustrating reduced *Akt1*^*−/−*^ myocardial volume. (E) *Akt1*^*−/−*^ fetuses displayed significantly lower weight at E16.5 compared to *Akt1*^*+/+*^ fetuses. RV, right ventricle; LV, left ventricle; +/+, *Akt1*^*+/+*^; +/*−*, *Akt1*^*+/−*^; *−*/*−*, *Akt1*^*−/−*^; *P < 0.05.

In order to examine myocardial morphology in high-resolution and 3D, ex vivo *μ*CT was used to validate in utero echocardiography. While the ventricular septum in E16.5 and E18.5 *Akt1*^*−*/*−*^ fetal hearts appeared thinned, CT examination of isolated hearts failed to expose any VSD's in either Akt1^*−*/*−*^ or Akt1^+/*−*^ hearts (Fig. 2A and B). The myocardium of *Akt1*^*−*/*−*^ fetal hearts appeared thinned and had a dilated morphology, as compared to *Akt1*^+/+^ fetal hearts. LV myocardial mass was significantly lower at E16.5 in *Akt1*^+/*−*^, and at E18.5 in *Akt1*^*+/−*^ and *Akt1*^*−/−*^ compared to *Akt1*^+/+^ fetal hearts. Total myocardial mass was also reduced at E16.5 and E18.5 Akt1^*−*/*−*^ fetuses compared to *Akt1*^+/+^ fetuses (Fig. 2C and D). Fetal *Akt1*^*−*/*−*^ body weight was significantly reduced, leaving LV mass index similar for all groups. Intrauterine growth retardation with reduced body weight has been previously described in *Akt1*^−/−^ mice (Cho et al. 2001; Yang et al. 2003; Plaks et al. 2010).

### Altered cardiac morphology, reduced amount of coronary blood vessels, and decreased number of myocardial capillaries observed in Akt1-deficient fetuses

**Figure 3:**
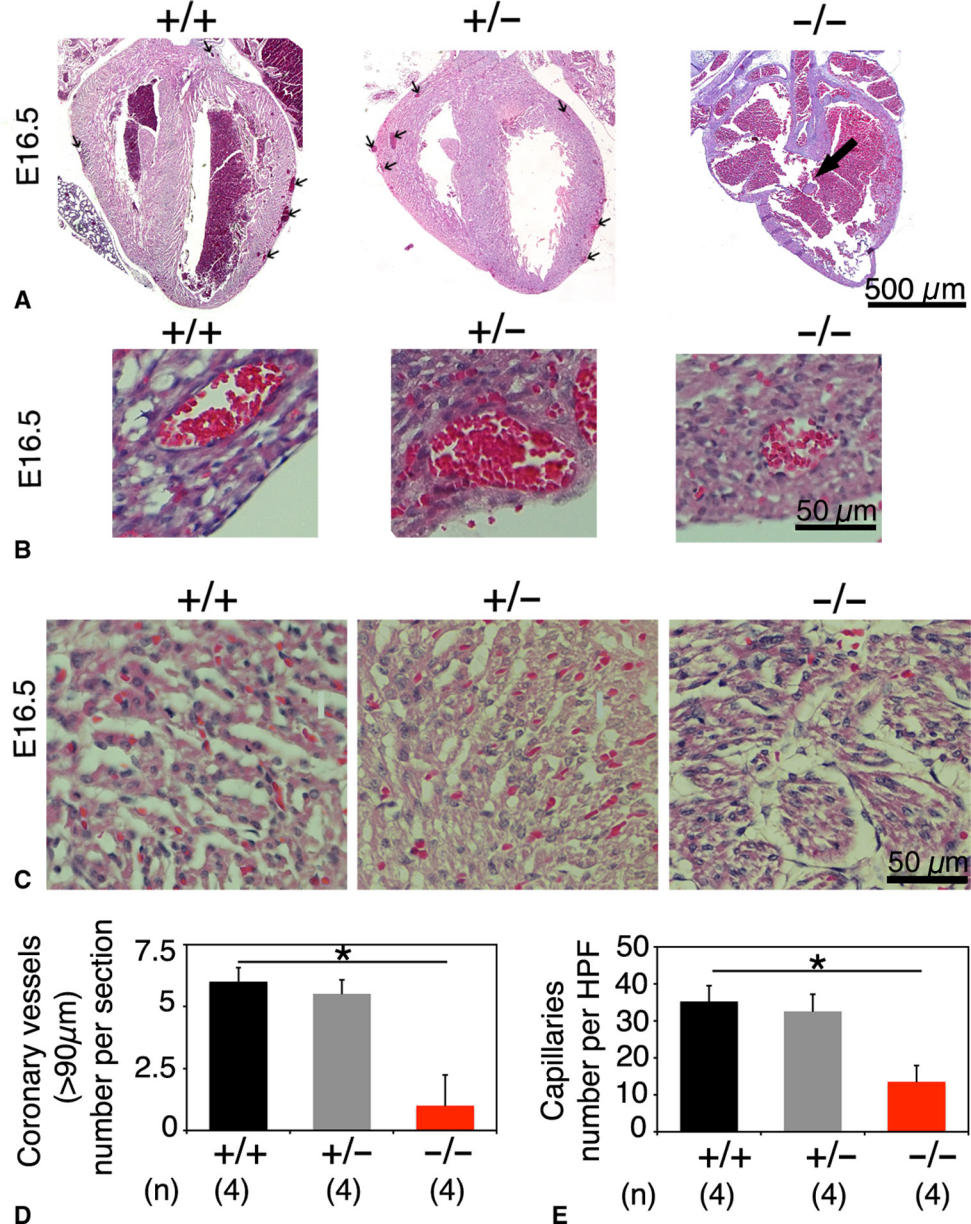
Histological analysis of E16.5 fetal *Akt1*^*+/+*^, *Akt1*^*+/−*^, and *Akt1*^*−/−*^ hearts, coronary blood vessels and myocardial capillaries. (A) Some *Akt1*^*−/−*^ fetal hearts displayed ventricular septum defects (VSDs; large arrow). Coronary vessels (>90 *μ*m) located beneath the epicardium were rarely present in *Akt1*^*−/−*^ fetal hearts (small arrows). (B) *Akt1*^*−/−*^ myocardial veins are smaller and have a thinner perivascular wall than *Akt1*^*+/+*^ veins (C) *Akt1*^*−/−*^ myocardium exhibited less myocardial capillaries than *Akt1*^*+/+*^ myocardium. (D) Quantification of the amount of coronary vessels (>90 *μ*m) per section. (E) Quantification of the amount of myocardial capillaries per high power field (HPF). (*P < 0.05).

Histological analysis of fetal hearts at E16.5 revealed in one of four *Akt1*^*−/−*^ fetuses a large VSD. All *Akt1*^*−/−*^ fetuses displayed a thin myocardium and a dilated morphology (Fig. 3A), consistent with prior histological data (Chang et al. 2010). Interestingly, we observed a reduced presence of coronary blood vessels larger than 90 *μ*m per cardiac section (Fig. 3A and D). Close-ups of the myocardial veins showed thinning of the perivascular wall in *Akt1*^*−/−*^ fetal myocardium (Fig. 3B). When looking at the highest magnification we could detect a decreased amount of myocardial capillaries per HPF in E16.5 *Akt1*^*−/−*^ fetuses compared to *Akt1*^*+/+*^ fetuses (Fig. 3C and E). The total amount of coronary blood vessels and myocardial capillaries in *Akt1*^*+/−*^ fetuses was not different from the amount in *Akt1*^*+/+*^ fetuses (Fig. 3).

### Neonatal *Akt1*^*−*/*−*^ mice displayed reduced survival and impaired myocardial angiogenesis

**Figure 4:**
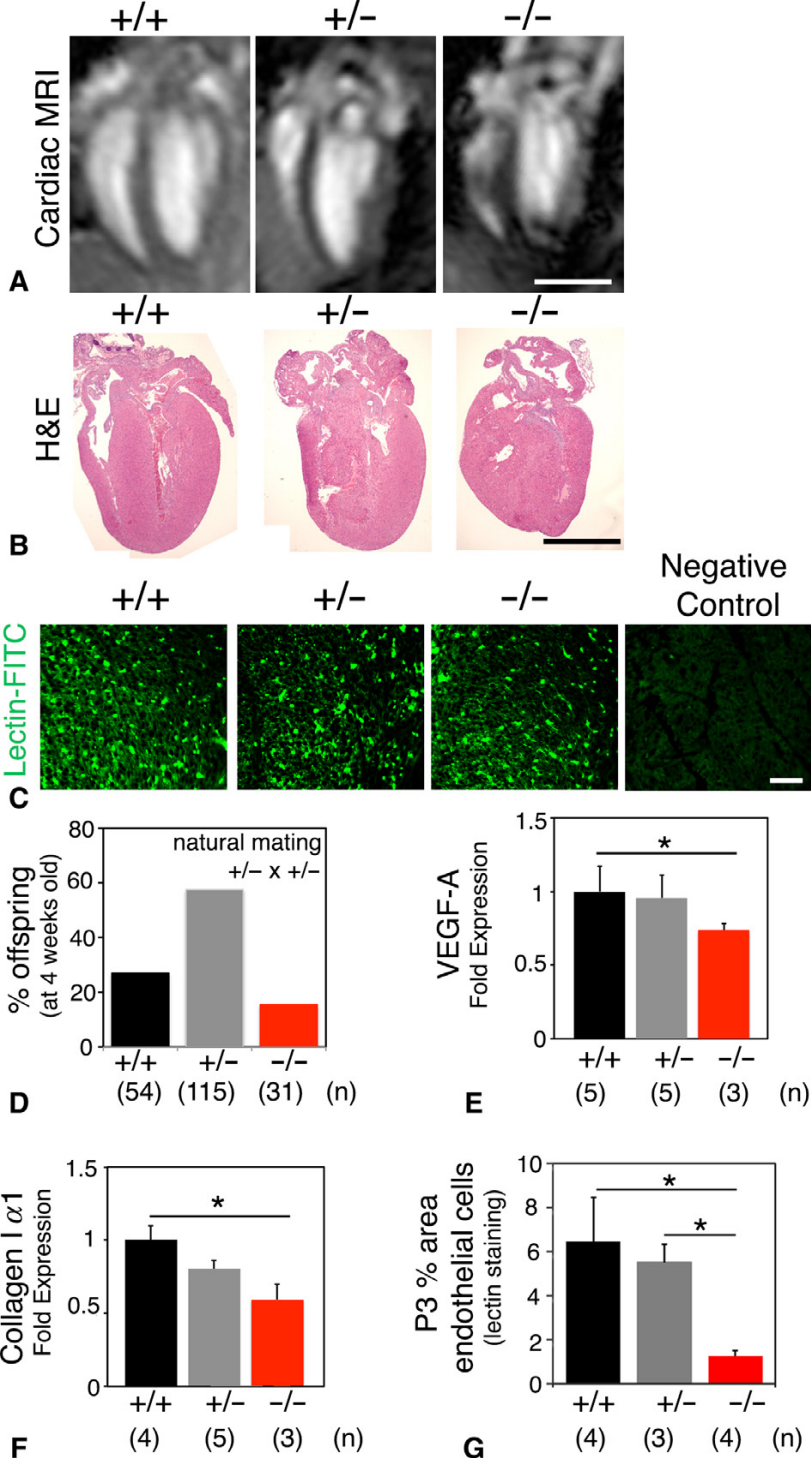
Akt1 deficiency at postnatal day 3 (P3) results in detectable changes in neonatal cardiac function, morphology and myocardial angiogenesis. (A) Neonatal cardiac MRI showing reduced cardiac size and altered morphology in *Akt1*^*−/−*^ mice (scale bar 1 mm). (B) H&E staining of neonatal hearts confirming reduced cardiac size and altered structure in *Akt1*^*−/−*^ mice (scale bar 1 mm). (C) Immunostaining using endothelial-cell specific lectin (green fluorescent) and negative control without lectin-FITC performed on myocardial sections of P3 mice; staining represent endothelial cells and endothelial cell precursors (scale bar 50 *μ*m). (D) Of 200 mice genotyped at 4 weeks old, 54 were *Akt1*^*+/+*^, 115 were *Akt1*^*+/−*^, and only 31 were *Akt1*^*−/−*^ (of which 62% survived to 4 weeks). (E) QRT-PCR of VEGF-A expression in P3 hearts, indicating reduced VEGF-A expression in *Akt1*^*−/−*^. (F) QRT-PCR of Collagen I*α*1 expression in P3 hearts, indicating reduced Collagen type I*α*1 expression in *Akt1*^*−/−*^. (G) Quantification of endothelial-cell specific lectin staining at the myocardium of P3; *Akt1*^*−/−*^ is characterized by a reduced amount of green fluorescent lectin-stained endothelial cells compared to *Akt1*^*+/+*^ and *Akt1*^*+/−*^. +/+, *Akt1*^*+/+*^; +/*−*, *Akt1*^*+/−*^; *−*/*−*, *Akt1*^*−/−*^; *P < 0.05).

Of 200 mice genotyped at 4 weeks old, 54 were *Akt1*^+/+^ (27% instead of the expected 25%), 115 were *Akt1*^+/*−*^ (57.5% instead of the expected 50%), and only 31 were *Akt1*^*−*/*−*^ (15.5%; fewer than the expected 25%; Fig. 4D). Although *Akt1*^*−*/*−*^ mice exhibited dilated LV morphology and thin myocardium throughout development, 62% of these *Akt1*^*−*/*−*^ mice survive to adulthood. *Akt1*^*−*/*−*^ mice exhibited enhanced peri-natal mortality as shown before (Cho et al. 2001; Chang et al. 2010; Plaks et al. 2010). Cine MRI of neonatal *Akt1*^*−*/*−*^ mice showed a trend of reduced cardiac function and reduced LV mass compared to *Akt1*^+/+^ littermates (Fig. 4A), consistent with previous echocardiographic measurements at P2 (Chang et al. 2010). Reduction in LV mass in neonatal *Akt1*^*−*/*−*^ mice is also visible on coronal H&E sections (Fig. 4B). To assess the level of angiogenesis in neonatal *Akt1*^*−*/*−*^ hearts, we measured the density of endothelial cells and their precursors in the myocardium. Lectin staining indicated a significantly lower quantity of endothelial cells in P3 *Akt1*^*−*/*−*^ myocardium compared to *Akt1*^+/*−*^ and *Akt1*^+/+^ (Fig. 4C and D). RT-PCR of neonatal hearts revealed reduced expression of VEGF-A in *Akt1*^*−*/*−*^ mice compared to *Akt1*^+/+^ and *Akt1*^+/*−*^ mice, as an indication for reduced angiogenesis (Fig. 4E). Moreover, reduced expression of collagen I*α*1, the most abundant collagen in connective tissue, was found in *Akt1*^*−*/*−*^ mice compared to *Akt1*^+/+^ and *Akt1*^+/*−*^ mice (Fig. 4F).

## Discussion

This in utero echocardiographic study showed morphological cardiac changes in *Akt1*^*−/−*^ fetuses resulting in abnormal mitral flow patterns and diastolic as well as systolic cardiac dysfunction. Ex vivo 3D μCT analysis revealed a reduced left ventricular volume, a dilated morphology with thinner fetal myocardium. Ex vivo histology confirmed altered cardiac morphology, and illustrated a reduced number of coronary blood vessels and myocardial capillaries in *Akt1*^*−*/*−*^ fetuses. In neonatal *Akt1*^*−*/*−*^ mice in vivo cardiac MRI and histology confirmed altered cardiac function and structure. Impaired myocardial angiogenesis with concomitant aberrant fibrinogenesis proceeded after birth in neonatal *Akt1*^*−*/*−*^ mice displaying reduced survival.

In this study we used multiple imaging modalities leading to unique and powerful datasets to generate simultaneous high-resolution anatomical and functional information. Multiparameter in utero echocardiographic analyses could associate a functional phenotype with the genotype of *Akt1*^*−*/*−*^ fetuses. PW Doppler analysis illustrated continuous reduced cardiac performance at late-stage fetuses. Altered fetal cardiac morphology appeared to contribute to fetal systolic and diastolic dysfunction. Only few fetuses displayed pericardial perfusions on echocardiography. These results are consistent with the cardioprotective properties previously reported for Akt (Matsui et al. 2001; Mangi et al. 2003). For fetal *Akt1*^*+/−*^ hearts, we demonstrated early-impaired cardiac function, an intermediate phenotype between *Akt1*^*+/+*^ and *Akt1*^*−/−*^ heart, underlining the intermediate phenotype reported before (Plaks et al. 2010; Vandoorne et al. 2010). Echocardiographic measures of wild-type *Akt1*^*+/+*^ fetuses from *Akt1*^*+/−*^ dams were consistent with a previous publication on wild-type C57bl/6 fetal hearts (Yu et al. 2008).

*Post mortem* histology and *μ*CT analysis of fetuses previously imaged by in utero echocardiography further linked the functional phenotype with a structural phenotype in *Akt1*^*−/−*^ fetuses. Dilated morphology, thinning of the myocardium, and an occasional VSD were observed in late-stage *Akt1*^*−/−*^ fetuses as previously also described in E14.5 fetuses (Chang et al. 2010). Although some major morphological changes were detected, in utero mortality was not identified at echocardiography. Given the reduced number of coronary vessels and myocardial capillaries, and thinning of the perivascular wall detected in *Akt1*^*−/−*^ fetuses on histological analyses, we speculate that impaired myocardial angiogenesis and coronary vasculogenesis may be causing *Akt1*^*−/−*^ fetal cardiac dysfunction. Residual and low levels of myocardial ischemia resulting from impaired microcirculatory formation may contribute to a morphology of ventricular wall thinning and LV dilation in Akt1^*−*/*−*^ fetuses similar to what is observed in chronic myocardial ischemia (Reese et al. 2002). Impaired development of the coronary vascular system can thus directly lead to impaired cardiac function (Reese et al. 2002).

At neonatal age, we reported reduced angiogenesis in *Akt1*^*−/−*^ hearts, with a smaller capillary network and decreased levels of VEGF-A. Remodeling of the extracellular matrix is essential for proper angiogenesis (Ivkovic et al. 2003). Expression of collagen I*α*1, a marker for fibrinogenesis, was found reduced in *Akt1*^*−*/*−*^ neonates compared to *Akt1*^+/+^ and *Akt1*^+/*−*^ neonatal mice, indicating disturbed remodeling of the extracellular matrix. Similar matrix abnormalities have been previously observed in *Akt1*^*−*/*−*^ mice (Somanath et al. 2007). Reduced angiogenesis and fibrinogenesis could further contribute to decreased survival in *Akt1*^*−*/*−*^ mice. In neonatal *Akt1*^*−/−*^ mice impaired cardiac function has previously been revealed using echocardiography, along with a thin myocardium and VSDs in the hearts of these mice (Chang et al. 2010). We underlined the same trend with a limited amount of animals using cardiac MRI at P3. Postnatal mortality and growth retardation in *Akt1*^*−/−*^ mice has been associated with placental insufficiency (Yang et al. 2003). However, when placental Akt1 was rescued by tetraploid complementation, growth retardation and phenotypical changes in the fetus proper remained (Plaks et al. 2010). Smaller neonatal hearts and growth retardation have also been shown in studies downregulating VEGF (Gerber et al. 1999). During fetal development of the heart, VEGF is expressed at the atrioventricular field forming the heart septa (Miquerol et al. 2000). Reduced VEGF levels might ascribe to VSDs detected in *Akt1*^*−/−*^ mice during early development. Yet also the disruption of other downstream targets of Akt1 such as GATA4, NFAT or MAPK (Sussman et al. 2011) during cardiac development has been assigned to cause VSDs (Ransom and Srivastava 2007). In embryonic *Akt1*^*−/−*^ hearts p38MAPK is substantially activated (Chang et al. 2010).

In human fetuses, VSD is the most common congenital heart disease, seen in 1.5–3.5 per 1000 live births, and accounting for 30% of all cardiac anomalies (Barboza et al. 2002). Interestingly, dilated cardiomyopathies are rare, accounting for 8–11% of fetal cardiovascular abnormalities (Pedra et al.^2002^). Dilated cardiomyopathy is the end result of various cardiac disease processes and is characterized by the dilation of cardiac chambers and the reduction in systolic function (Pedra et al.^2002^). It remains unclear whether mutations in Akt1 gene play a role in human cardiac disease. The *Akt1*^*−*/*−*^ model might present an interesting model for studying growth retardation, VSDs, and cardiomyopathy in utero.

The primary limitation of this study is our use of systemically Akt1 ablated mice, and thus the observed phenotype could be attributed to the contribution of multiple cell types. Additionally, we could not perform serial imaging of the mice during early development, as genotyping was required and there are very limited noninvasive methods described to landmark fetuses and neonatal mice (Kulandavelu et al. 2006; Avni et al. 2012). In addition, the lateral spatial resolution of the ultrasound system was limited to 115 *μ*m, which given the small fetal heart size in mice (1–2 mm), may have limited in utero detection of VSDs. Finally, we did not assess umbilical and peripheral flows. *Akt1*^*−/−*^ fetuses have a smaller and less perfused placenta and have phenotypical changes in the vascular bed of several organs (Yang et al. 2003; Plaks et al. 2010; Vandoorne et al. 2010). It is possible that the observed phenotype is complicated by differences in placental and peripheral perfusion in the *Akt1*^*−/−*^ fetuses.

In conclusion, in utero echocardiography appeared to be a powerful tool to characterize cardiac function in late-stage fetal mice with loss of Akt1. The hearts of growth-retarded *Akt1*^*−/−*^ fetuses exhibited abnormal blood flow patterns at the mitral valve and reduced contractile function. We were not only able to link these functional changes to structural alterations like dilated cardiomyopathy and VSDs using ex vivo methods, but we have also shown reduced amount of myocardial capillaries and coronary vessels in *Akt1*^*−/−*^ fetal hearts. These changes continued after birth and may account for reduced survival of *Akt1*^*−/−*^ neonates. Researching fetal cardiovascular function as well as delineation of the underlying pathogenesis might improve the prenatal and perinatal management providing prognostic information and improving outcomes of affected pregnancies.
